# Antirheumatic Drugs against COVID-19 from the Perspective of Rheumatologists

**DOI:** 10.3390/ph14121256

**Published:** 2021-12-02

**Authors:** Mai Kawazoe, Mari Kihara, Toshihiro Nanki

**Affiliations:** 1 Division of Rheumatology, Department of Internal Medicine, School of Medicine, Faculty of Medicine, Toho University, Tokyo 143-8541, Japan; mai.kawazoe@med.toho-u.ac.jp; 2 Department of Rheumatology, Tokyo Medical and Dental University, Tokyo 113-8519, Japan; kihara-mari@pmda.go.jp

**Keywords:** SARS-CoV-2, COVID-19, cytokine storm, antirheumatic drugs, immunosuppressants

## Abstract

Coronavirus disease 2019 (COVID-19) remains a global threat to humanity. Its pathogenesis and different phases of disease progression are being elucidated under the pandemic. Active viral replication activates various immune cells and produces large amounts of inflammatory cytokines, which leads to the cytokine storm, a major cause of patient death. Therefore, viral inhibition is expected to be the most effective early in the course of the disease, while immunosuppressive treatment may be useful in the later stages to prevent disease progression. Based on the pathophysiology of rheumatic diseases, various immunomodulatory and immunosuppressive drugs are used for the diseases. Due to their mechanism of action, the antirheumatic drugs, including hydroxychloroquine, chloroquine, colchicine, calcineurin inhibitors (e.g., cyclosporine A and tacrolimus), glucocorticoids, cytokines inhibitors, such as anti-tumor necrosis factor-α (e.g., infliximab), anti-interleukin (IL)-6 (e.g., tocilizumab, sarilumab, and siltuximab), anti-IL-1 (e.g., anakinra and canakinumab) and Janus kinase inhibitors (e.g., baricitinib and tofacitinib), cytotoxic T lymphocyte-associated antigen 4 blockade agents (e.g., abatacept), and phosphodiesterase 4 inhibitors (e.g., apremilast), have been tried as a treatment for COVID-19. In this review, we discuss the mechanisms of action and clinical impact of these agents in the management of COVID-19.

## 1. Introduction

COVID-19, caused by severe acute respiratory syndrome coronavirus 2 (SARS-CoV-2), was initially identified in Wuhan, China in December 2019. Since then, the disease has spread globally and resulted in an ongoing unprecedented pandemic. Although the majority of COVID-19 patients exhibit mild disease manifestations, approximately 5–15% develop severe symptoms, including acute respiratory disease syndrome (ARDS), systemic shock, and multi-organ failure [1,2,3]. The mechanisms underlying severe COVID-19 have not yet been elucidated in detail; however, accumulating evidence suggests the dysregulated and excessive host’s immune response to viral infection, called the cytokine storm [1,4].

**Figure 1:**
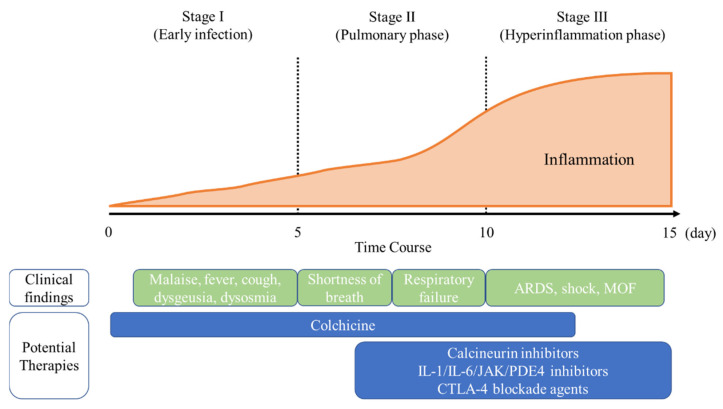
Classification of COVID-19 states and potential therapies. The figure illustrates the three escalating phases of COVID-19 progression with associated symptoms and potential phase-specific therapies. ARDS, acute respiratory distress syndrome; CTLA, cytotoxic T lymphocyte-associated antigen; IL, interleukin; JAK, Janus kinase; MOF, multiple organ failure; PDE, phosphodiesterase.

Although the pathogenesis of COVID-19 currently remains unclear, a three-stage classification system has been proposed, with increasing severity reflecting differences in clinical findings, responses to therapy, and clinical outcomes (Figure 1). Stage I (early infection) begins when SARS-CoV-2 entry, and the infection is established. Patients may or may not manifest non-specific symptoms (i.e., fever, malaise, a sore throat, dry cough, dysgeusia, and dysosmia). In Stage II (pulmonary phase), viral multiplication and localized inflammation in the lungs are common. During this stage, patients may develop viral pneumonia with fever and cough, and hypoxia. Stage III (hyperinflammation phase) is characterized by a hypersensitive response of the immune system. In this stage, systemic inflammatory markers are elevated and characterized by a hypercoagulable state that may progress to multiple organ failure. Therefore, antiviral treatment may be the most effective in the early stage of COVID-19, and immunosuppressive treatment in the later stage of the disease [1,4].

Approved therapeutics for the treatment of COVID-19 remain limited. In addition to the use of specific antivirals, several drugs for the treatment of rheumatic diseases have so far been used under crisis conditions based on at least some mechanistic rationale. A large number of randomized controlled trials (RCTs) are ongoing, with several now reporting their findings. The findings of cohort studies and small- and moderate-sized case series provide important information on the utility of currently available immunomodulatory therapies as well. The aim of this review is to summarize the mechanisms of action of antirheumatic drugs, including immunomodulatory and immunosuppressive drugs, for the potential treatment of COVID-19, and the rationale and evidence for its efficacy in the health emergency we are currently facing.

## 2. Mechanisms of SARS-CoV-2 Infection

**Figure 2:**
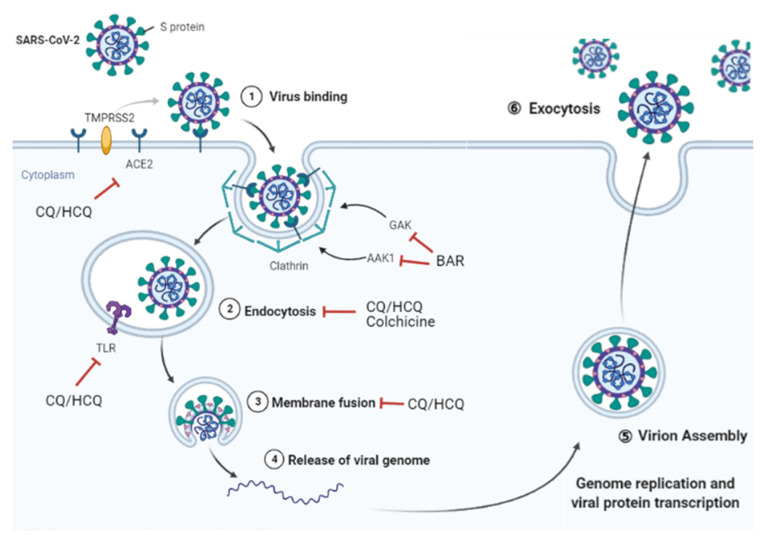
Overview of the mechanism of SARS-CoV-2 infection. The spike (S) protein on the surface of SARS-CoV-2 binds to ACE2 expressed on host cells, such as nasal and bronchial epithelial cells and pneumocytes. TMPRSS2 present on the host cell surface subsequently primes the S protein and promotes endocytosis-induced viral entry into the cell. The virus is carried into the endosome and uncoated by fusing with the endosome membrane, which releases its genomic RNA into the cytoplasm. Genomic RNA replication and protein synthesis occur in the ribosome, forming viral particles and releasing them extracellularly. Active viral replication activates various immune cells and produces large amounts of inflammatory cytokines. CQ and HCQ inhibits the glycosylation of ACE2, which may interfere with the binding of SARS-CoV-2 to the cell receptor. CQ and HCQ decrease acidity in endosomes and inhibit the fusion of SARS-CoV-2 to host cell membranes, and also interfere with TLR signaling by changing local pH. Colchicine may affect clathrin-mediated endocytosis. BAR binds AAK1 and GAK, the identified regulators of endocytosis, and reduces viral entry. ACE2, angiotensin-converting enzyme II; AAK1, AP2-associated protein kinase 1; BAR, baricitinib; CQ, Chloroquine; GAK, cyclin G-associated kinase; HCQ, hydroxychloroquine; TLR, Toll-like receptor; TMPRSS2, transmembrane protease serine 2.

A more detailed understanding of the mechanisms underlying SARS-CoV-2 infection allows for the identification of targets for novel therapeutic agents to prevent or treat the disease. An overview of these mechanisms is found in Figure 2. SARS-CoV-2 is a single-stranded RNA-enveloped virus. In the early stage of infection, the S (spike) protein on the surface of SARS-CoV-2 binds to the angiotensin-converting enzyme II (ACE2) expressed in host cells, such as nasal and bronchial epithelial cells and pneumocytes, and particularly alveolar epithelial type II cells (Figure 2). The transmembrane protease serine 2 (TMPRSS2) present on the host cell surface subsequently primes the S protein and promotes endocytosis-induced viral entry into cells. The virus is carried into the endosome and uncoated by fusing with the endosome membrane, which releases genomic RNA into the cytoplasm. Genomic RNA replication and protein synthesis occur in the ribosome, forming viral particles in the endoplasmic reticulum-Golgi intermediate compartment and releasing them extracellularly.

Active viral replication activates various immune cells and produces large amounts of inflammatory cytokines, which leads to the cytokine storm. Elevated serum levels of inflammatory cytokines, including interleukin (IL)-2, IL-4, IL-6, IL-7, IL-10, tumor necrosis factor (TNF), and interferon (IFN)-γ, have been reported in severe COVID-19 patients [5,6,7,8]. The cytokine storm, by excess activation of immune cells, exhibits systemic hyperinflammation, which in turn, causes ARDS, multiple organ failure, and ultimately patient death [9]. There are various triggers of the cytokine storm, such as viral and bacterial infections, autoimmune diseases, malignancies, and drugs. Among them, cytokine storm, which can occur in patients with rheumatic diseases such as adult-onset Still’s disease, systemic juvenile idiopathic arthritis, and systemic lupus erythematosus, is called macrophage activation syndrome (MAS), and we rheumatologists sometimes encounter it. Severe COVID-19 and MAS demonstrated similarities in the clinical features of the cytokine storm, such as fever and respiratory failure due to ARDS, and dysregulated cytokine profiles [10]. For treatment with MAS, we use glucocorticoids and immunosuppressants.

**Figure 3:**
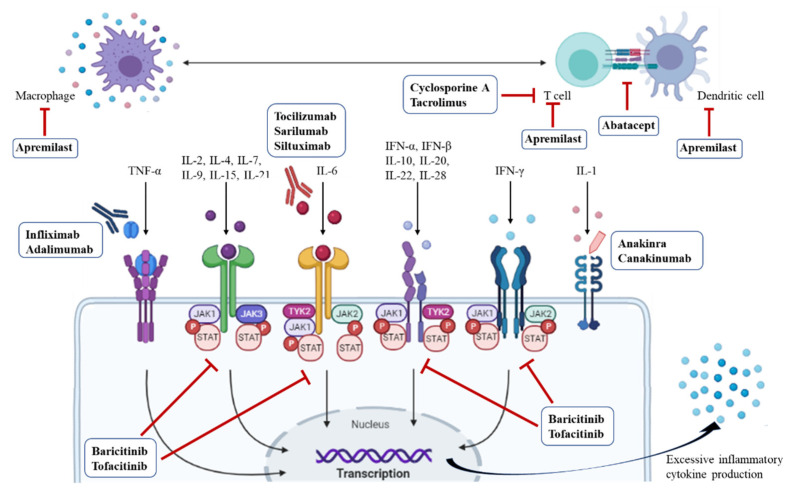
Schematic representation of sites of action of antirheumatic drugs. Janus kinases (JAK1, JAK2, JAK3, and TYK2) are activated by extracellular stimuli, including cytokines, and phosphorylate downstream STAT proteins, which translocate to the nucleus and activate target genes to produce inflammatory cytokines. IFN, interferon; IL, interleukin; JAK, Janus kinase; P, phosphoric acid; STAT, signal transducer and activator of transcription; TNF, tumor necrosis factor; TYK, tyrosine kinase.

Once the cytokine storm is caused, by an excessive immune response, antiviral therapy alone is not sufficient; anti-inflammatory and immunosuppressive therapies must be combined. Therefore, stage-specific therapeutic strategies that intervene in the progression of COVID-19 may be considered based on the previously explained three stages of pathogenesis. Viral inhibition is expected to be most effective in the early stages of the disease, while anti-inflammatory and immunosuppressive therapies may be effective in the later stages of COVID-19 to prevent disease progression. To date, a wide variety of antirheumatic drugs have been evaluated for their ability to target the viral and host immune responses (Table 1). Drugs for the treatment of rheumatic diseases have been the focus of attention as a potential therapy since the beginning of the COVID-19 epidemic, and observational studies and clinical trials have been conducted on a wide variety of drugs. We therefore reviewed the mechanisms of action of promising repurposed antirheumatic drugs for COVID-19 in Figure 2 and Figure 3, and, below, evaluate the published clinical experiences of them.

**Table 1:** Characteristics of Antirheumatic Drugs Under Evaluation for the Treatment of COVID-19.

Drug	Mechanism of Action	Dosage Used in Some Clinical Trials	Potential Efficacy Reported in Clinical Trials	Recommendations
Chloroquine Hydroxychloroquine	Inhibit viral entry, endocytosis, and production of cytokines	800 to 1600 mg p.o. 1 to 3 divided doses on the first day, and then 200 to 800 mg in 1 to 2 divided doses daily for 5 to 21 days	None	WHO recommend against administering chloroquine or hydroxychloroquine, except in a clinical trial
Colchicine	Inhibit endocytosis	0.5 mg p.o. twice daily for 3 days, then once daily for 27 days	Administration as soon as the diagnosis reduces the risk of disease aggravation and the resulting number of hospitalization Improve points on a 7-grade clinical status scale	
Cyclosporine A	Suppress the T cell response Inhibit the pro-inflammatory cytokines	1–2 mg/kg/day p.o. divided into two doses for 7 days	Improve outcomes and reduce mortality, mainly in patients with moderate to severe disease	
Tacrolimus	Suppress the T cell response	Dose to achieve plasma levels of 8–10 ng/mL p.o.	Not reported	
Glucocorticoids	Suppress inflammation and excessive immune response	dexamethasone (6 mg daily for up to 10 days)	Reduce 28-day mortality	The Panel recommends using dexamethasone in hospitalized patients with COVID-19 who require supplemental oxygen
Tocilizumab	Suppresses cytokine storms by IL-6 inhibition	8 mg/kg i.v. (maximum dose 800 mg) in combination with dexamethasone	Improve 90-day survival Reduce organ support-free days Reduce time to discharge	The Panel recommends concomitant use of dexamethasone in recently hospitalized patients who are exhibiting rapid respiratory decompensation
Sarilumab		400 mg i.v.		The Panel recommends its use only when tocilizumab is not available or is not feasible to use
Siltuximab		11 mg/kg i.v.	Not yet reported	
Anakinra	Suppress the cytokine storm, reduce endothelial dysfunction and microvascular alteration by inhibiting IL-1	200 mg i.v. twice daily for 3 days, 100 mg i.v. twice daily on day 4, and 100 mg i.v. on day 5	Reduce mortality at day 28 and the need for mechanical ventilation	
Canakinumab		450 mg for body weight 40–59 kg, 600 mg for 60–80 kg or 750 mg for >80 kg i.v.	Improve oxygenation Decrease serum CRP levels	
Baricitinib	Reduce viral entry Inhibit proinflammatory intracellular signals of some cytokines by inhibiting JAK1 and JAK2	4 mg p.o. per day for 14 days or until hospital discharge	Reduce a median time to recovery by 1 day Accelerate improvement in clinical status Reduce 28-day mortality	The Panel recommends concomitant use with dexamethasesone in recently hospitalized patients receiving high-flow oxygen therapy or non-invasive ventilation
Tofacitinib	Inhibit proinflammatory intracellular signals of some cytokines by inhibiting JAK1 and JAK3	10 mg p.o. twice daily on day 1, followed by 5 mg twice daily on day 2–5	Not yet reported	
Infliximab	Suppress inflammation by inhibiting TNF-α	5 mg/kg i.v.	Reduced mortality	
Abatacept	Inhibit T cell activation and suppress the production of inflammatory cytokines	10 mg/kg i.v. (maximum dose 1000 mg)	Not yet reported	
Apremilast	Prevent the overproduction of inflammatory cytokines by inhibiting PDE4	30 mg p.o. twice daily for 14 days	Antipyretic effects Improve oxygenation	

COVID-19, coronavirus disease 2019; IL, interleukin; i.v., intravenous; JAK, Janus kinase; PDE, phosphodiesterase; p.o., per os; the Panel, the COVID-19 Treatment Guidelines Panel of the National Institutes of Health; TNF, Tumor Necrosis Factor; WHO, World Health Organization.

## 3. Pharmacotherapy

### 3.1. Chloroquine (CQ) and Hydroxychloroquine (HCQ)

CQ and HCQ (Aralen^®^ and Plaquenil^®^), an analogue of CQ, are used to treat rheumatic diseases, such as rheumatoid arthritis (RA) and systemic lupus erythematosus (SLE), in addition to malaria. The chemical structure of HCQ differs from that of CQ by a hydroxyl group at the end of the N-ethyl side chain [11,12]. Clinically, HCQ has less toxicities, including QTc prolongation and retinopathy, and fewer drug-drug interactions than CQ [12]. These drugs decrease acidity in endosomes and inhibit the fusion of SARS-CoV-2 to host cell membranes [13]. This prevents viral genomic RNA from being released into the cytoplasm and inhibits the proliferation of the virus. In addition, SARS-CoV-2 has been found to use the same cell entry receptor, ACE2, as SARS-CoV [14]. CQ inhibits the glycosylation of ACE2, which may interfere with the binding of SARS-CoV to cell receptors [15]. Therefore, the findings of in vitro studies suggested that CQ and HCQ both prevent the release of the viral genome [16]. CQ and HCQ also exhibit immunomodulatory activities, which may synergistically enhance their antiviral effects in vivo. Moreover, CQ and HCQ interfere with Toll-like receptor (TLR) signaling by changing local pH [12]. TLR signaling pathways stimulate cytokine production, and CQ and HCQ have been found to inhibit the production of various cytokines, including IL-6, TNF, and IFN-γ, by mononuclear cells [17].

Due to their mechanisms of action, CQ and HCQ were expected to become therapeutic agents for COVID-19, and early studies on their administration received widespread attention despite limited conclusive evidence. On 28 March 2020, the Food and Drug Administration (FDA) issued the emergency use of CQ and HCQ for patients hospitalized with COVID-19. However, the FDA subsequently revoked its emergency use on 15 June 2020 because findings from the SOLIDARITY trial and RECOVERY trial both demonstrated that HCQ had no effect on the mortality of hospitalized patients with COVID-19 [18,19,20].

The SOLIDARITY trial is a large, international, open-label RCT conducted by the World Health Organization (WHO) that evaluated the effects of HCQ on in-hospital mortality in hospitalized patients with at least one of the following: a clinical assessment (evidence of rales/crackles in an examination) and blood oxygen saturation [SpO_2_] of 94% or less in room air or acute respiratory failure requiring mechanical ventilation and/or supplemental oxygen [19]. Death by day 28 occurred in 104 out of 947 patients receiving HCQ and 84 out of 906 patients receiving usual care alone (11.0% versus [vs.] 9.3%; rate ratio 1.19; 95% confidence interval [CI], 0.89 to 1.59; *p* = 0.23). The findings of this trial demonstrated that HCQ had negligible or no effects on not only mortality, but also ventilation requirement and length of hospital stay. The RECOVERY trial is also a large, open-label RCT on hospitalized patients with COVID-19 in the United Kingdom that compared 1561 patients who were given HCQ to 3155 patients who received the standard of care alone [20]. The findings obtained showed no significant differences in 28-day mortality rates (27.0% in the HCQ group vs. 25.0% in the standard of care group; rate ratio, 1.09; 95% CI, 0.97 to 1.23; *p* = 0.15). The proportion of patients being discharged alive within 28 days from hospital was lower in the HCQ group (59.6% vs. 62.9%; rate ratio, 0.90; 95% CI, 0.83 to 0.98), and also those who were not using ventilators at baseline were more likely to initiate invasive ventilators or to be dead in the HCQ group (30.7% vs. 26.9%; risk ratio, 1.14; 95% CI, 1.03 to 1.27).

Combination therapy with HCQ and azithromycin compared to HCQ alone has also been evaluated, but open-label RCTs found no improvement of clinical outcomes among hospitalized patients with mild to severe COVID-19 [21,22].

Numerous studies have reported more adverse events, such as QT prolongation, in patients who received HCQ with or without azithromycin than in those who received the standard of care [23]. However, whether and to what extent HCQ increases the risk of cardiotoxicity remain unclear [24]. Because azithromycin and HCQ both have long half-lives (68 h and 30–60 days, respectively) [25,26], caution should be taken even when the two drugs are used sequentially rather than in combination [27].

Recent studies examined the efficacy of the prophylactic administration of CQ and HCQ. A randomized, double-blind, placebo-controlled clinical trial on HCQ prophylaxis in 132 health care workers in the United States was conducted [28]. The incidence of SARS-CoV-2 infection during the eight weeks of treatment did not significantly differ in participants that received HCQ and placebo (6.3% vs. 6.6%; *p* > 0.99). A randomized, double-blind, placebo-controlled trial testing HCQ as postexposure prophylaxis was also conducted in the United States and Canada [29]. It enrolled 821 asymptomatic adults who had household or occupational contact with individuals with confirmed COVID-19 for at least 10 min at a distance of 6 feet or less and were randomized to receive either placebo or HCQ within 4 days of exposure. The percentage of subjects who presented with symptoms compatible with COVID-19 within 14 days was not significantly different between those who received HCQ and those who received placebo (11.8% vs. 14.3%), with an absolute difference of −2.4 percentage points (95% CI, −7.0 to 2.2; *p* = 0.35). Therefore, there was no clinical benefit of HCQ in pre- or post-exposure prophylaxis.

Based on the findings of a systematic review and network meta-analysis that collected data from 30 RCTs on 10,921 patients, in December 2020, the WHO strongly recommended against the use of HCQ or CQ in addition to usual care for the treatment of patients with COVID-19 regardless of disease severity or the duration of symptoms [24]. Similarly, based on the findings of a systematic review and meta-analysis of 3 clinical trials on 2444 patients that examined the efficacy of HCQ as prophylaxis (pre- and post-exposure) [30], the WHO announced in March 2021 that it strongly recommended against the use of HCQ as a prophylactic drug for COVID-19 [31].

### 3.2. Colchicine

Colchicine (Colcrys^®^) is an oral anti-inflammatory drug indicated for the treatment of gout, familial Mediterranean fever, and Behçet’s disease [32,33]. Colchicine is considered to exert its effects mainly by inhibiting microtubule polymerization and neutrophil chemotaxis and the suppression of IL-1β and IL-18 release; however, a recent study demonstrated that the anti-inflammatory effects of colchicine were mainly attributable to the inhibition of Nod-like receptor family pyrin domain-containing 3 (NLRP3) inflammasomes [34]. The findings obtained from experimental models demonstrated that NLRP3 inflammasomes may be activated and triggered by different SARS-CoV-2 proteins [35,36,37] and, subsequently, may be involved in the pathophysiological cascade of acute lung injury and/or ARDS [38]. Therefore, colchicine may influence clathrin-mediated endocytosis. This process is partly mediated by microtubule remodeling [39], and may delay the viral infection of cells [40]. Colchicine may also be utilized to treat COVID-19 as a major inhibitor of NLRP3 inflammasomes, and, based on these perspectives, several RCTs are being conducted to assess the potential of colchicine to improve outcomes in patients with COVID-19.

In an open-label RCT in Greece, 105 patients hospitalized with COVID-19 were randomized to receive colchicine or the standard of care alone [41]. The mean time to deterioration by 2 points on a 7-grade clinical status scale, which means the clinical condition requiring non-invasive/invasive mechanical ventilation or death, was 18.6 days the in the standard of care group and 20.7 days in the colchicine group (Log-rank *p* = 0.03). COLCORONA is a randomized, double-blind, placebo-controlled trial conducted on non-hospitalized patients with COVID-19 with at least one high-risk criterion, such as old age or complications [42]. An analysis of 4159 PCR-confirmed COVID-19 patients revealed that colchicine reduced hospitalizations by 25%, the need for mechanical ventilation by 50%, and deaths by 44% for 30 days after enrollment.

These RCT findings indicated the significant benefit of colchicine for improving the outcomes of both hospitalized and non-hospitalized patients with COVID-19. Based on these findings, the administration of colchicine to patients at risk for complications once they are diagnosed with COVID-19 may reduce the risk of severe disease and consequently reduce the number of hospitalizations. Moreover, positive findings from the COLCORONA trial demonstrated that colchicine may be an effective drug for the treatment of non-hospitalized patients.

### 3.3. Calcineurin Inhibitors

Cyclosporine A (CsA; Neoral^®^, Sandimmune^®^) is a calcineurin inhibitor isolated from the fungi *Tolypocladium inflatum* and *Cylandrocarpon lucidum*, obtained from Norwegian soil in 1976. Tacrolimus (TAC; Prograf^®^) is also a calcineurin inhibitor isolated from the actinomycete *Streptomyces tsukubaensis*, obtained from Japanese soil in 1984. They are used as an immunosuppressant for patients with rheumatic diseases, such as RA, SLE, and idiopathic inflammatory myopathy [43,44]. The complexes cyclophilin-CsA and FK506-binding protein-TAC bind to phosphatase calcineurin, which inhibits the dephosphorylation of nuclear factor of activated T cells (NFAT) [45]. This interferes with the entry of NFAT into the T-cell nucleus and further suppresses cytokine production, such as IL-2, thereby suppressing the T-cell response. CsA is also an immunomodulator of the innate immune response, suppressing the pro-inflammatory cytokines such as IL-1β, IL-12, and TNF, while promoting the anti-inflammatory cytokine such as IL-10 [46]. In addition, CsA and TAC have been shown to exhibit antiviral activity in vitro and inhibit the replication of several coronaviruses, including SARS-CoV and MERS-CoV [47,48,49,50].

An open-label, non-randomized study was conducted to compare the clinical efficacy of CsA with glucocorticoids to glucocorticoids in 209 patients with COVID-19 pneumonia [51]. Mortality rates within 28 days were 22% and 35% in the CsA and the control group (*p* = 0.02), respectively, for all patients, and 24% and 48.5% for patients with moderate to severe disease (*p* = 0.001). In patients with moderate to severe disease, there was a greater cumulative clinical improvement in the CsA group (Nelson-Aalen curve, *p* = 0.001, Log-rank test). These findings suggested that the addition of CsA to glucocorticoids improve outcomes and reduce mortality, mainly in patients with moderate to severe COVID-19.

Although there have been no published clinical reports on the efficacy of TAC to treat COVID-19, a European multicenter study on 243 liver transplant recipients recently reported that the use of TAC was associated with an increased survival probability in COVID-19 patients [52]. Therefore, TAC is expected to exert therapeutic effects against COVID-19, and a RCT on severe COVID-19 patients is underway (ClinicalTrials.gov number, NCT04341038) [53].

### 3.4. Glucocorticoids

Glucocorticoids are widely used to treat a number of diseases, including rheumatic diseases, and improve patient outcomes due to their potent anti-inflammatory properties. Patients with severe COVID-19 may develop systemic inflammatory responses that lead to lung injury and multiple organ failure. Glucocorticoids may play a role in preventing or mitigating these deleterious effects and in suppressing lung inflammation due to their anti-fibrotic properties, particularly in the advanced stages of COVID-19 [54].

In a meta-analysis that aggregated seven randomized trials and included data from 1703 critically ill patients with COVID-19, the administration of dexamethasone was found to reduce 28-day all-cause mortality rates more than usual care or placebo [55]. The largest trial in this meta-analysis was the RECOVERY trial, a multicenter, randomized, open-label trial on hospitalized patients with COVID-19 in the United Kingdom [56]. In this study, 2104 patients receiving dexamethasone (at a dose of 6 mg, once daily) for up to 10 days were compared with 4321 patients receiving the standard of care. In the dexamethasone group, the mortality rate within 28 days was lower in patients on ventilation than in the standard care group (29.3% vs. 41.4%; rate ratio, 0.64; 95% CI, 0.51 to 0.81) and lower in patients on oxygen than in the standard care group (23.3% vs. 26.2%; rate ratio, 0.82; 95% CI, 0.72 to 0.94), but not in patients who did not require respiratory support (17.8% vs. 14.0%; rate ratio,1.19; 95% CI, 0.92 to 1.55). Largely based on data from this RECOVERY trial, the COVID-19 Treatment Guidelines Panel of the National Institutes of Health (the Panel) recommend the use of dexamethasone in hospitalized patients with COVID-19 who require supplemental oxygen [57]. RCTs using various formulations such as prednisolone, methylprednisolone, and hydrocortisone have all had small sample sizes, and evidence of their efficacy has not been established [55,58,59,60,61]. Therefore, the Panel recommended the use of other glucocorticoids such as prednisone, methylprednisolone, and hydrocortisone in equivalent doses if dexamethasone is not available.

In an RCT of hospitalized patients with severe COVID-19 pneumonia, 34 patients received intravenous methylprednisolone (mPSL) pulses (250 mg daily for 3 days), and 28 patients received standard of care alone [62]. The mortality rate was significantly lower in the mPSL group (5.9% vs. 42.9%; *p* < 0.001). Intubation rate was also significantly lower in the mPSL group (8.8% vs. 32.1%), and survival was significantly prolonged (HR, 0.29; 95% CI, 0.15–0.56; *p* < 0.001 by Log-rank test). However, the level of evidence is low because this RCT was single-blind and the mPSL dosage was different from the common dosage (1000 mg per day, intravenous for 3 days), and the results of future studies are awaited.

There are theoretical concerns that glucocorticoids may slow viral clearance. In patients with other viral respiratory infections, glucocorticoids have been reported to delay viral clearance and/or worsen clinical outcomes [63,64,65]. Therefore, at the beginning of the COVID-19 epidemic, the WHO did not recommend glucocorticoid use for COVID-19 induced pneumonia. However, in the RECOVERY trial, the incidence of death, not only due to COVID-19 but also due to other infections, was not higher in the dexamethasone group than in the standard of care group [56]. Based on this result, the WHO guideline recommendations were updated in September 2020 to recommend glucocorticoids for patients with severe and critical COVID-19. A recent study of patients with moderate to severe COVID-19 found no association between glucocorticoids use and viral clearance rates [66]. Moreover, some studies suggest that viral shedding in SARS-CoV-2 is higher in the early stage of COVID-19 and declines thereafter [67,68]. Due to discrepancies in the findings of observational studies and the absence of clinical trial data, some Panel members consider the co-administration of dexamethasone and remdesivir to patients who have recently been placed on mechanical ventilation until more conclusive evidence becomes available [69].

Clinicians should be aware of side effects (e.g., hyperglycemia, secondary infections, psychiatric effects, and avascular necrosis) in COVID-19 patients receiving dexamethasone. Long-term use of glucocorticoids may increase the risk of reactivation of latent infections such as hepatitis B virus and tuberculosis. The risk of reactivation of latent infections when dexamethasone is administered for only 10 days is not clear at this time [70,71]. However, when initiating dexamethasone, appropriate screening and treatment should be considered to reduce the risk of reactivation of fulminant hepatitis. Precautions regarding the risk of reactivation of occult infections should also be taken in the use of the immunosuppressive drugs described below.

### 3.5. IL-6 Inhibitors

IL-6 is one of the key mediators of autoimmunity, inflammation, and the cytokine storm [72]. Several observational and in vitro studies have shown that IL-6 is a crucial cytokine associated with the severity of COVID-19 and mortality [7,8]. In a meta-analysis, the mean IL-6 level in severe COVID-19 patients was 2.9 fold higher than in non-severe patients, and elevated IL-6 was correlated with adverse clinical outcomes such as intensive care unit (ICU) admission, ARDS, and death [73]. Moreover, recent studies implicated the cytokine storm in the severity of COVID-19 and poor outcomes [5,73,74,75]. Based on these findings, the pharmacological blockade of IL-6 receptor is expected to reduce the severity of COVID-19 and may be a therapeutic target for managing dysregulated host responses in patients with COVID-19.

Several IL-6 inhibitors are commercially available for clinical use. Tocilizumab (Actemra/RoActemra^®^) is a recombinant humanized monoclonal antibody that binds to the IL-6 receptor. It is the first anti-IL-6 receptor biologic approved for RA [76]. The FDA has also approved tocilizumab for the treatment of giant cell arteritis, polyarticular/systemic juvenile idiopathic arthritis, interstitial lung disease associated with systemic sclerosis, and chimeric antigen receptor T cell-induced cytokine-release syndrome [77]. Sarilumab (KEVZARA^®^) is a recombinant human monoclonal antibody that antagonizes IL-6 receptors, and has been approved for the treatment of RA by the FDA. Siltuximab (SYLVANT^®^) is a recombinant human-mouse chimeric monoclonal antibody that directly binds to IL-6 and is FDA-approved for the treatment of multicentric Castleman’s disease in HIV and HHV-8-negative patients [78].

To date, many observational studies on and case reports of COVID-19 patients receiving IL-6 inhibitors, mostly tocilizumab, have demonstrated improved clinical outcomes. However, because evidence of drug efficacy is important for the choice of COVID-19 therapeutics, we herein discuss the findings of studies, mainly previously reported and ongoing RCTs, that help to identify the potential effectiveness of these drugs. CORIMUNO-TOCI is an open-label RCT of 130 hospitalized patients in France with moderate or severe COVID-19 pneumonia, who required at least 3 L/min of oxygen but did not require ventilation or admission to the ICU [79]. Patients in the tocilizumab group had a lower rate of death or need for ventilation (non-invasive or mechanical) on day 14 compared with patients in the usual care group (24% vs. 36%, median posterior hazard ratio [HR] 0.58; 90% credible interval [CrI], 0.33 to 1.00). However, there was no difference in mortality rates on day 28 between the two groups (adjusted HR, 0.92; 95% CI, 0.33 to 2.53). The COVACTA trial [80] was the first global, randomized, double-blind, placebo-controlled study of 438 hospitalized patients with severe COVID-19 pneumonia, including approximately 38% mechanically ventilated patients. It failed to achieve the primary endpoint of improvement in clinical status at day 28, which was assessed using a 7-category ordinal scale [81] based on the requirement for intensive care, ventilators, and supplemental oxygen (odds ratio, 1.19; 95% CI, 0.81 to 1.76; *p* = 0.36). There was also no difference in mortality at day 28 (19.7% vs. 19.4% with a difference of 0.3%; 95% CI, −7.6% to 8.2%; *p* = 0.94). It should be noted that the rate of concomitant use of glucocorticoids was low in these trials.

The EMPACTA trial [82] is a global, randomized, double-blind, placebo-controlled trial that was conducted on 389 hospitalized patients with COVID-19 pneumonia not requiring ventilation. The primary endpoint, the percentage of progress to mechanical ventilation or death by day 28, was 44% lower in patients who received tocilizumab plus the standard of care compared with placebo plus the standard of care (12.0% vs. 19.3%; HR, 0.56; 95% CI, 0.32–0.97: *p* = 0.04 by Log-rank test). However, there was no significant difference in all-cause mortality at day 28 (10.4% vs. 8.6%; weighted difference 2.0%, 95% CI, −5.2 to 7.8). The REMAP-CAP trial [83] is a multinational RCT of 803 critically ill patients with suspected or confirmed COVID-19, admitted to the ICU and receiving respiratory or cardiovascular organ support. Patients were randomly assigned to receive IL-6 receptor antagonists (353 patients received tocilizumab and 48 received sarilumab) or standard treatment (402 patients) within 24 h of admission to the ICU, with a median hospitalization of 1.2 days (interquartile range [IQR], 0.8 to 2.8). The median number of days without organ support, the primary endpoint, were 10 (IQR, −1 to 16), 11 (IQR, 0 to 16), and 0 (IQR, −1 to 15) in the tocilizumab, sarilumab, and control groups, respectively. In comparisons with the control group, the median adjusted odds ratios were 1.64 (95% CrI, 1.25 to 2.14) in the tocilizumab group and 1.76 (95% CrI, 1.17 to 2.91) in the sarilumab group, with posterior probabilities of superiority greater than 99.9% and 99.5%, respectively. In-hospital mortality rates were 28.0% in the tocilizumab group, 22.2% in the sarilumab group, and 35.8% in the control group. Tocilizumab and sarilumab were found to be effective in all secondary endpoints, including 90-day survival, times to ICU admission and hospital discharge, and improvements in the WHO ordinal scale [84] at 14 days. It is important to note that the majority of patients in these trials were enrolled after the announcement of the dexamethasone findings from the RECOVERY trial [56]; therefore, they were treated with glucocorticoids at enrollment or within the subsequent 48 h. In the RECOVERY trial, 4116 patients with SpO_2_ less than 92% or receiving oxygen and CRP greater than 7.5 mg/dL were randomized to tocilizumab or standard of care [85]. The 28-day mortality rate was significantly lower in the tocilizumab group (31% vs. 35%; RR 0.85; 95% CI 0.76 to 0.94; *p* = 0.0028). In this study, 82% of patients received concomitant glucocorticoids, and results of subgroup analysis also suggest that the combination of tocilizumab and dexamethasone improves mortality, especially in patients requiring noninvasive ventilation and high-flow oxygen.

In an industry-sponsored phase III trial of sarilumab in patients with severe or critical COVID-19 pneumonia requiring mechanical ventilation, manufacturers announced in July 2020 that sarilumab failed to meet its primary and key second endpoints [86]. Subsequently, the sarilumab trial in the United States was subsequently terminated. Trials conducted outside the United States were continued, and the findings of a global randomized trial on 420 patients with COVID-19 pneumonia requiring oxygen or intensive care that compared sarilumab (161 patients in the 200 mg group, 173 patients in the 400 mg group) and placebo (86 patients) in addition to the standard of care were announced [87]. On day 29, the median time to at least a 2-point improvement from baseline on the 7-point scale was 12.0 days for placebo versus 10.0 days for 200 mg sarilumab and 10.0 days for 400 mg sarilumab, with no difference. There was also no difference in the proportions of patients alive: 92% in the placebo group compared with 90% in the 200 mg sarilumab group (difference, −1.7; *p* = 0.63) and 92% in the 400 mg sarilumab group (difference, 0.2; *p* = 0.85).

Based on the findings from the REMAP-CAP trial, the UK Government announced in January 2021 that it will begin the widespread administration of tocilizumab or sarilumab to patients with COVID-19 in the ICU across the UK, in combination with dexamethasone. The NHS England subsequently published the Interim Clinical Commissioning Policy for tocilizumab on February [88]. The Panel also updated its recommendations for the use of tocilizumab in certain populations of patients with COVID-19 in March 2021 [89]. The Panel currently recommends the use of tocilizumab with concomitant dexamethasone (plus remdesivir) in recently hospitalized patients (within 3 days of hospitalization) receiving high-flow oxygen therapy or non-invasive ventilation who have rapidly increasing oxygen requirements or increased inflammatory markers, because several trials have shown clinical benefit of tocilizumab in patients receiving tocilizumab plus glucocorticoids. For patients requiring invasive mechanical ventilation or extracorporeal membrane oxygenation who are within 24 h of ICU admission, the combination of tocilizumab and dexamethasone is recommended. Because there is more extensive evidence of efficacy for tocilizumab than for sarilumab, sarilumab is recommended only when tocilizumab is not available or is not feasible to use [57].

Remdesivir is an antiviral medicine that inhibits replication of SARS-CoV-2 [13] and has been approved and authorized for temporary use to treat COVID-19 in approximately 50 countries worldwide. Therefore, the findings of the global phase III randomized, double-blind, multicenter REMDACTA study in hospitalized patients with severe COVID-19 pneumonia comparing tocilizumab with remdesivir and placebo with remdesivir were awaited; however, it was announced that the primary endpoint of improvement in time to hospital discharge at day 28 was not achieved [90]. The trial also did not meet key secondary endpoints, which included the likelihood of death, likelihood of progression to mechanical ventilation or death, and the clinical status. These results suggested that IL-6 inhibitors alone might be insufficient to suppress the inflammatory phase of COVID-19.

Siltuximab was reported to reduce mortality in an open-label cohort study of 30 patients with COVID-19 pneumonia requiring ventilatory support [91]. The Panel recommends against the use of siltuximab for the treatment of COVID-19, except in clinical trials [57].

To summarize the findings of multiple trials, tocilizumab and sarilumab may reduce the need for respiratory and cardiovascular organ support, ICU-level care and time to discharge, and hospital mortality in patients with severe COVID-19. However, there is no clear evidence because differences in mortality attributable to tocilizumab were not observed across RCTs [92], in contrast to previous observational studies and case reports. The reasons for differences across the studies are unclear, but may be due to various degrees of respiratory dysfunction in the patients enrolled in each trial, racial and ethnic differences in the populations studied, and differences in the frequency of glucocorticoid use (more frequent use in the EMPACTA trial and REMAP-CAP trial). Further data from ongoing RCTs are needed to correctly assess the benefits of using IL-6 inhibitors to manage COVID-19, including the timing and route of administration and different patient populations, which will provide a more detailed understanding of whether this therapy is effective.

### 3.6. IL-1 Inhibitors

IL-1 is a pro-inflammatory cytokine that is secreted by macrophages, monocytes, and dendritic cells after TLRs are activated by viruses, leading to the formation of inflammasomes. IL-1 can be divided into two major types: IL-1α and IL-1β. IL-1β is released from monocytes, macrophages, and neutrophils, and mediates neutrophils recruitment, inflammation, and fever in viral infections [93]. High IL-1 levels have been detected in the serum of patients with COVID-19 [7,94], and, thus, IL-1 inhibitors are currently used to treat COVID-19 and are also expected to be applied to the cytokine storm. The efficacy of IL-1 blockade with anakinra against the cytokine storm has already been reported in 44 pediatric patients with MAS associated with rheumatic and non-rheumatic conditions [95]. Moreover, inhibition of IL-1 has been associated with reduced endothelial dysfunction and microvascular changes [96], which appear to be important in COVID-19-related thromboembolic events [97].

Anakinra (KINERET^®^) is a recombinant human IL-1 receptor antagonist that can be intravenously or subcutaneously administered and is the first IL-1 blocking biologic to be produced. Anakinra inhibits the pro-inflammatory effects of IL-1 by blocking the binding of both IL-1α and IL-1β to the IL-1 receptor. It is approved by the FDA to treat RA and cryopyrin-associated periodic syndromes (CAPS), an autoinflammatory disease that often occurs in the neonatal period [93]. Canakinumab (ILARIS^®^), an IL-1 inhibitor, is a high-affinity, fully humanized monoclonal anti-IL-1β antibody approved for the treatment of CAPS, systemic juvenile idiopathic arthritis, and other periodic fever syndromes [98].

A retrospective cohort study in Italy included hospitalized COVID-19 patients having respiratory failure (a ratio of the partial pressure of oxygen to the fraction of inspired oxygen (PaO_2_/FiO_2_) of 300 mmHg or less) and high inflammation (serum CRP concentration of 100 mg/L or higher or a ferritin concentration of 900 ng/mL or higher) [99]. Among 392 patients, 275 did not receive an IL inhibitor, while 62 received anakinra. In multivariate analysis, patients who received anakinra had a significantly lower risk of death compared to those who did not receive IL inhibitors (HR, 0.45; 95% CI, 0.20 to 0.99; *p* = 0.047). A prospective, open-label interventional study of 69 hospitalized patients with severe COVID-19 pneumonia also reported improved clinical outcomes [100]. Forty-five patients received anakinra and 24 were in the control group. Ventilation was required in 14 patients (31%) in the anakinra group and 18 patients (75%) in the control group (*p* < 0.001). In-hospital deaths were 13 (29%) in the anakinra group and 11 (46%) in the control group (*p* = 0.082). Twenty-five patients (63%) in the anakinra group and 6 patients (27%) in the control group were able to wean off supplemental oxygen (*p* = 0.008).

Several RCTs are currently in progress to evaluate the efficacy of anakinra in patients with COVID-19, and the findings of one of these trials was reported. In a multicenter, open-label RCT conducted in France, patients with mild to moderate COVID-19 pneumonia requiring oxygenation at least 3 L/min by mask or nasal cannula, not requiring ventilation and ICU admission at hospital admission, and with a serum CRP concentration of higher than 25 mg/L were included [101]. The proportion of patients who died or required non-invasive ventilation or mechanical ventilation by day 4 was 36% (21 out of 59 patients) in the anakinra group and 38% (21 out of 57 patients) in the usual care group (the median posterior absolute risk difference, −2.5%; 90% CrI, −17.1 to 12.0). On day 14, 28 patients (47%; 95% CI, 33 to 59) in the anakinra group required mechanical or non-invasive ventilation or died, compared with 28 patients (51%; 95% CI, 36 to 62) in the usual care group. These results suggested that anakinra did not improve the outcome of patients with mild to moderate COVID-19 pneumonia. The REMAP-CAP, described in the “IL-6 inhibitors” section, is an adaptive platform trial evaluating the effect of various interventions to improve the outcomes of patients admitted to the ICU with COVID-19 pneumonia [102]. The median organ support-free days was 0 days (IQR, −1 to 15 days) in the 365 patients who received anakinra versus 0 days (IQR, −1 to 15 days) in the 418 who received standard therapy (OR, 0.99; 95% CrI, 0.74 to 1.35). In-hospital survival was 60.3% in the anakinra group and 63.1% in the control group (OR, 0.97; 95% CrI, 0.66 to 1.40).

The efficacy of canakinumab was evaluated in a randomized, double-blind, placebo-controlled trial conducted in the United States and Europe, which enrolled hospitalized patients with hypoxic COVID-19 pneumonia who did not require invasive ventilation [103]. The survival without the need for invasive mechanical ventilation between days 3 and 29 was 198 of 223 patients (88.8%) in the canakinumab group and 191 of 223 patients (85.7%) in the placebo group (OR 1.39; 95% CI, 0.76 to 2.54; *p* = 0.29).

Therefore, the Panel announced that there is not sufficient evidence to recommend the use of anakinra for the treatment of COVID-19. In addition, the Panel does not recommend the use of canakinumab for the treatment of COVID-19, except in clinical trials.

### 3.7. Janus Kinase (JAK) Inhibitors

Basic science has identified a variety of intracellular pathways that regulate normal and abnormal immune responses [104], one of which is the JAK/STAT (JAK/signal transducer and activator of transcription) pathway. This pathway mediates signal transduction from extracellular stimuli such as cytokines, growth factors, and hormones to the cell nucleus [105,106,107]. JAK is a tyrosine kinase (TYK), an enzyme that specifically phosphorylates tyrosine residues in proteins, and a specific JAK is bound to each cytokine receptor. When a cytokine binds to a receptor, STAT, which is a transcription factor, is phosphorylated by JAK, dimerized, and then transported into the nucleus to regulate transcription. Four JAK members (JAK1, JAK2, JAK3, and TYK2) and 7 STAT members (STAT1, STAT2, STAT3, STAT4, STAT5A, STAT5B, and STAT6) have been identified to date, and each combination transmits various signals induced by different cytokines. For example, innate antiviral responses via type I IFN (IFN-I) are mediated by JAK1/TYK2, while type II IFN (IFN-II) signaling is mediated by JAK1/JAK2. IL-6 transduces signaling via complexes of JAK1, JAK2, and TYK2 [107,108].

JAK inhibitors were initially launched as a treatment for RA. JAK inhibitors are low-molecular-weight products that inhibit intracellular signal transduction by competitively binding to the ATP-binding site of JAK in cells and inhibiting phosphorylation [107,108]. Five JAK inhibitors are currently marketed for RA: tofacitinib, a selective inhibitor of JAK1 and JAK3, upadacitinib and filgotinib, selective inhibitors of JAK1, peficitinib, an inhibitor of pan-JAK, and baricitinib, a selective inhibitor of JAK1 and JAK2 [109]. Among them, tofacitinib and baricinitib have been applied for use as therapeutic drugs for COVID-19.

As discussed earlier, increased concentrations of serum cytokines and chemokines correlated with the severity of COVID-19 and adverse clinical outcomes [7]. Several cytokines that were previously reported to be elevated in severe COVID-19 patients use intracellular signaling pathways mediated by JAKs [8,107,110]. Therefore, the interruption of this pathway by JAK inhibitors may block multiple cytokines at the same time, which may represent an attractive strategy for preventing hyperinflammation, or the so-called cytokine storm, caused by SARS-CoV-2 [111,112].

On the other hand, a point of concern regarding the use of JAK inhibitors in COVID-19 is interference with endogenous IFN and with the immune response to the virus [108]. IFN-I (IFN-α and IFN-β) plays a major role in the innate immune response, preventing viral replication in the early stages of infection [113]. The activation of IFN-II (IFN-γ) signaling leads to the up-regulation of several IFN-stimulated genes with the ability to rapidly kill viruses within infected cells [114]. However, a recent study revealed that IFN-I and, to a lesser extent, IFN-II up-regulated the expression of ACE2 in several human cell lines, including upper airway epithelial cells and primary bronchial cells [115]. Moreover, acute clinical deterioration in moderate COVID-19 patients requiring inpatient care may be the result of increased inflammation due to elevated cytokine levels, such as type I IFNs and IL-6 [7,8], all of which transmit their signals through the JAK-STAT pathway. The protective antiviral effects of IFN may be less important in this phase. Another concern is the occurrence of thromboembolic events. Some cases of venous thromboembolism have been reported in patients with RA treated with JAK inhibitors [116]. An increasing number of studies have reported that coagulation abnormalities are the hallmark of severe COVID-19 infection, with more prominent abnormalities developing as patients progress from a severe to critical state. The direct attack of SARS-CoV-2 on endothelial cells may potentially contribute to coagulopathy in COVID-19 [117,118]. In addition, regardless of the initial triggering factors, fibrin deposition in the alveolar lumen due to activation of coagulation and inhibition of fibrinolysis is important in the pathophysiology of ARDS [119]. Indeed, increased D-dimer has been noted to be a predictor of ARDS incidence and mortality [120,121]. On the other hand, D-dimer level was significantly lower in patients treated with baricitinib plus glucocorticoids than in those treated with glucocorticoids alone [122]. This reflects the protective effects of baricitinib on the pulmonary endothelium, which may contribute to improvement of the respiratory status of patients treated with baricitinib.

Baricitinib (Olumiant^®^) is a JAK inhibitor that is currently approved for the treatment of moderate to severe active RA and atopic dermatitis. By selectively inhibiting JAK1 and JAK2, baricitinib intracellularly inhibits the pro-inflammatory signals of several cytokines, such as IL-6, IL-12, IL-23, and IFN-γ. Baricitinib also binds to AP2-associated protein kinase 1 (AAK1) and cyclin G-associated kinase (GAK), regulators of endocytosis, and may reduce viral entry, as well as inflammation [123]. The clinically significant study was the ACTT-2 trial, a multinational, double-blind, randomized, placebo-controlled trial evaluating baricitinib in hospitalized patients with COVID-19 pneumonia with SpO_2_ below 94% or requiring oxygenation [124]. All patients received remdesivir and either baricitinib or placebo. Recovery was defined as improvement to a condition that did not require oxygen or could be discharged, with a median time to recovery of 7 days (95% CI, 6 to 8) in 515 patients receiving baricitinib, and 8 days (95% CI, 7 to 9) in 518 controls (RR, 1.16; 95% CI, 1.01 to 1.32; *p* = 0.03). In the subgroup of patients who received high-flow oxygen or non-invasive ventilation at baseline, it was 10 days in the baricitinib group compared to 18 days in the control group (RR, 1.51; 95% CI, 1.10 to 2.08). The odds of improvement in the clinical status by day 15 was higher in the baricitinib group than in the control group (OR, 1.3; 95% CI, 1.0 to 1.6). Mortality rates at day 28 were 5.1% in the baricitinib group and 7.8% in the control group (HR, 0.65; 95% CI, 0.39 to 1.09). No significant differences were observed in the frequency of pulmonary embolism (5 vs. 2 patients, respectively) or deep vein thrombosis (11 vs. 9 patients, respectively) between the two groups. Since the difference of reduced recovery time was small in the overall cohort and was considered clinically inadequate, the Panel currently recommends the use of baricitinib with concomitant dexamethasone (plus remdesivir) in recently hospitalized patients (within 3 days of hospitalization) receiving high-flow oxygen therapy or non-invasive ventilation who have rapidly increasing oxygen requirements or increased inflammatory markers [57]. We were unable to evaluate the therapeutic efficacy of baricitinib in addition to or compared with glucocorticoids as standard therapy for severe or critical COVID-19 pneumonia, because 10.9% of patients in the baricitinib group and 12.9% of patients in the placebo group received glucocorticoids in this study. The ACTT-4 trial (ClinicalTrials.gov Identifier: NCT04640168) is currently in progress to compare the clinical efficacy of “baricitinib and remdesivir” vs. “dexamethasone and remdesivir”.

The COV-BARRIER trial, a double-blind, randomized, placebo-controlled phase III trial evaluated the efficacy of baricitinib in hospitalized patients with COVID-19 pneumonia, or active and symptomatic COVID-19 [125]; 764 patients were assigned to receive baricitinib and the standard of care, and 761 patients were assigned to receive placebo and the standard of care. The primary endpoint, the percentages of patients progressing to high-flow oxygen, noninvasive ventilation, invasive mechanical ventilation, or death by day 28, did not differ between the baricitinib and placebo groups (27.8% vs. 30.5%; OR 0.85; 95% CI, 0.67 to 1.08; *p* = 0.18). All-cause 28-day mortality was 8.1% in the baricitinib group and 13.1 % in the placebo group, with a 38.2% reduction for baricitinib (HR 0.57; 95% CI, 0.41 to 0.78; nominal *p* = 0.002). In the subgroup of patients who received high-flow oxygen or non-invasive ventilation at baseline, it was 17.5% in the baricitinib group compared to 29.4% in the placebo group (HR 0.52; 95% CI, 0.33 to 0.80; nominal *p* = 0.007). Based on these findings, the Panel recommends the use of baricitinib with concomitant dexamethasone (plus remdesivir) in recently hospitalized patients (within 3 days of hospitalization) receiving high-flow oxygen therapy or non-invasive ventilation who have rapidly increasing oxygen requirements or increased inflammatory markers.

Since there are no studies directly comparing baricitinib and tocilizumab in the treatment of COVID-19, there is not enough evidence to recommend either drug. A phase III clinical trial (ClinicalTrials.gov Identifier: NCT04693026) is ongoing to evaluate the efficacy of “baricitinib and remdesivir” vs. “tocilizumab and remdesivir” for the treatment of severe ARDS caused by COVID-19.

Tofacitinib is an inhibitor of JAK1 and JAK3, with partial selectivity to JAK2 and, thus, may effectively block IL-2, IL-4, and IL-6. In contrast to baricitinib, we did not find any reports of tofacitinib being administered to patients with COVID-19. However, a cohort study (ClinicalTrials.gov identifier: NCT04750317) that investigated whether tofacitinib is effective at reducing the risk of mechanical ventilation and/or death in 414 patients with moderate to severe COVID-19 pneumonia has already been completed and the findings obtained are awaited. Tofacitinib is currently undergoing a phase II trial mainly intended to assess its safety and efficacy in hospitalized patients with COVID-19 pneumonia. It is designed as a multicenter study with 260 participants and is randomized and double-blinded with a placebo control (ClinicalTrials.gov Identifier: NCT04469114). Another similar phase II trial with 60 participants is ongoing to assess improvements in the clinical outcomes of patients with moderate SARS-CoV-2 infection (ClinicalTrials.gov Identifier: NCT04415151).

### 3.8. TNF Inhibitors

TNF plays a key role in almost all acute inflammatory reactions by inducing oxidative stress and inflammation, and it is a pro-inflammatory cytokine that is intimately involved in excessive inflammation [126]. It has also been found to mediate the transition from pulmonary inflammation to fibrosis [127]. As a pro-inflammatory cytokine, TNF has been implicated in lung and vascular tissue damage, ARDS, and coagulopathy [128,129].

TNF inhibitors (infliximab, etanercept, adalimumab, golimumab, and certolizumab pegol) are used in the management of several autoimmune inflammatory diseases, such as RA, inflammatory bowel disease, and ankylosing spondylitis. A TNF inhibitor suppresses inflammation and also reduces D-dimer level and pro-thrombin fragments [130]; therefore, a TNF inhibitor may also attenuate COVID-19-induced thrombosis. A study on mice reported that TNF significantly contributed to acute lung injury and that the neutralization of TNF activity or loss of TNF receptors provided protection against SARS-CoV-induced morbidity and mortality [131]. Serum TNF levels were previously found to be markedly elevated in patients with COVID-19 and positively correlated with disease severity and death [6], suggesting the potential of TNF inhibitor for COVID-19. Interestingly, a lower odd of hospitalization was reported in COVID-19 patients treated with TNF inhibitors for rheumatic diseases (OR, 0.40; 95% CI, 0.19 to 0.81) [132].

Clinical experiences with TNF inhibitor for COVID-19 have been reported with infliximab, a monoclonal antibody against TNF, only. A case series of 7 patients with severe COVID-19 treated with infliximab demonstrated a decrease in serum IL-6 levels and reduced mortality rates (35% in the control group vs. 14% in the infliximab group) [133]. A prospective, single center, phase II trial evaluating the efficacy of infliximab in 17 hospitalized patients with severe or critical COVID-19 pneumonia (ClinicalTrials.gov Identifier: NCT04425538) has already been completed, and the findings are awaited. A large randomized, placebo-controlled trial evaluating the efficacy of infliximab in patients with moderate to severe COVID-19 is currently ongoing (ClinicalTrials.gov Identifier: NCT04593940). Moreover, a cohort study comparing tocilizumab vs. tocilizumab/infliximab in patients with COVID-19-associated cytokine storm syndrome is underway (ClinicalTrials.gov Identifier: NCT04734678).

### 3.9. Cytotoxic T Lymphocyte-Associated Antigen 4 (CTLA-4)-Ig

T lymphocytes play an important role in the pathogenesis of RA by producing pro-inflammatory cytokines, promoting the formation of ectopic lymphoid structures and neovascularization in synovial tissues, promoting the production of autoantibodies by B cells, and activating synovial cells and osteoclasts. Abatacept (Orencia^®^) is a soluble fusion protein that consists of the extracellular domain of human CTLA-4, and has been approved for the treatment of moderate to severe active RA [134]. Abatacept binds CD80 and CD86 on antigen-presenting cells, and blocks interactions with CD28 on T cell and CD80 or CD86, T cell co-stimulation. In vitro, abatacept has been found to decrease T-cell proliferation and inhibit the production of TNF, IFN-γ, and IL-2.

Abatacept may also be effective for COVID-19 by suppressing T cell activation [135]. There is currently no information on abatacept being administered to patients with COVID-19; however, two clinical trials have been registered for abatacept therapy in COVID-19 patients. A phase II, randomized, double-blind placebo-controlled study is ongoing to evaluate the efficacy of intravenous abatacept in hospitalized COVID-19 participants with respiratory compromise (ClinicalTrials.gov identifier: NCT04472494). A large randomized, placebo-controlled trial on patients with moderate to severe COVID-19 is also underway to evaluate the efficacy of abatacept (ClinicalTrials.gov Identifier: NCT04593940).

### 3.10. Phosphodiesterase 4 (PDE4) Inhibitors

PDE4 is an enzyme that decomposes cyclic adenosine monophosphate (cAMP) into AMP. The degradation of cAMP regulates the production of pro-inflammatory and anti-inflammatory cytokines and cell proliferation [136]. The inhibition of PDE4 exerts multiple anti-inflammatory effects in a variety of cells due to the wide distribution of PDE4 expression: macrophages (down-regulation of TNF and IL-12, up-regulation of IL-10), dendritic cells (inhibition of antigen presentation), T-helper cells (down-regulation of Th1 proliferation and release of IFN-γ, IL-2, IL-4, IL-13, IL-17, and IL-22), B cells (attenuation of antibody production), and epithelial cells (down-regulation of inflammatory mediators and enhancements in barrier integrity).

Apremilast (Otezla^®^) is an oral first-in-class PDE4 inhibitor that was approved for the treatment of moderate to severe psoriasis, psoriatic arthritis, and oral ulcers associated with Behçet’s disease. It has been found to effectively inhibit pro-inflammatory cytokines, such as TNF, IFN-γ, IL-2, IL-12, IL-17, and IL-23, in inflammatory cells, both in vitro and in vivo [136,137]. Due to the role of PDE4, its inhibitors may attenuate the cytokine storm in COVID-19 through the upstream inhibition of pro-inflammatory molecules and the regulation of the pro-inflammatory/anti-inflammatory balance [138]. To date, only one case series has been reported for COVID-19 pneumonia treated with apremilast [139]. All four patients had severe pulmonary involvement (respiratory rate greater than 30 breaths per minutes or SpO_2_ less than 93% at rest or PaO_2_/FiO_2_ less than 300 mmHg, and/or pulmonary infiltrates greater than 50% on chest X-ray). Apremilast rapidly restored respiration and improved gas exchange in patients unresponsive to supportive care.

Two clinical trials are currently underway to administer apremilast to patients with COVID-19 (ClinicalTrials.gov Identifier: NCT02735707, NCT04590586). One of these is REMAP-COVID, which uses the same core design as REMAP-CAP [140]. REMAP-COVID has expanded enrollment to include all hospitalized patients with confirmed or clinically diagnosed COVID-19, and added an investigational drug group targeting COVID-19. Apremilast has been evaluated as immunomodulatory therapy in this trial since October 2020, and its findings are awaited.

## 4. Conclusions

We reviewed clinical reports of antirheumatic drugs for COVID-19. Most of them are expected to improve the cytokine storm caused by excess immune response and inflammation that can occur in severe COVID-19. However, some drugs, like chloroquine/hydroxychloroquine, were initially expected to be effective, but later denied the efficacy. Conversely, several drugs, such as glucocorticoids, were later recommended for use. Since various clinical trials on the efficacy of antirheumatic drugs in the treatment of COVID-19 are still ongoing, we must keep up to date. Although findings on several antirheumatic drugs suggest therapeutic benefits in patients with COVID-19, only dexamethasone, tocilizumab, sarilumab, and baricitinib are recommended in guidelines in some countries. Further clinical studies are required to ascertain the therapeutic potential of each antirheumatic drug as well as the appropriate timing of their administration. Novel antiviral drugs such as remdesivir are also expected, and antirheumatic drugs may be better used in combination with antiviral drugs. Further treatment strategies may be needed.
